# Diagnostic and Management Pitfalls of SGLT2 Inhibitor‐Associated Ketoacidosis in the ICU: Lessons Following Cardiac Surgery and GLP‐1 Agonist Interaction

**DOI:** 10.1155/crcc/4896065

**Published:** 2026-08-06

**Authors:** Ana Paula García Pérez, Damián Gutiérrez-Zárate, Karina Rosas-Sánchez, Alejandro Molina Romo, Manuel Nicolás Poblano

**Affiliations:** ^1^ Valle de México University, Santiago de Querétaro, Mexico; ^2^ Department of Intensive Care and Coronary Care Unit, Hospital Angeles Centro Sur, Santiago de Querétaro, Mexico; ^3^ Department of Cardiovascular Surgery, Hospital Angeles Centro Sur, Santiago de Querétaro, Mexico; ^4^ Department of Internal Medicine, Hospital Angeles Centro Sur, Santiago de Querétaro, Mexico

**Keywords:** critical care, diabetic ketoacidosis, SGLT2i-associated ketoacidosis, sodium-glucose cotransporter 2 inhibitor

## Abstract

Sodium‐glucose cotransporter 2 inhibitors (SGLT2i) are cornerstones in the treatment of Type 2 diabetes (T2D), but their use is associated with diabetic ketoacidosis (DKA), a potentially life‐threatening complication in the critical care setting. We present two cases of male patients with T2D who developed SGLT2i‐associated ketoacidosis under different stressors: the first following coronary artery bypass graft (CABG) surgery and the second after the addition of semaglutide to his therapeutic regimen. Both patients presented with high anion gap metabolic acidosis, ketonuria, and plasma glucose levels < 200 mg/dL. Management required intravenous insulin protocols and glucose supplementation to reverse ketosis. This report emphasizes the need for preoperative suspension protocols and close monitoring of changes in combination therapy to avoid diagnostic delays in the intensive care unit.

## 1. Introduction

SGLT2i have transformed the management of Type 2 diabetes (T2D) and have demonstrated additional benefits in heart failure and chronic kidney disease, supported by robust evidence across multiple clinical scenarios [1]. However, potential complications such as SGLT2i‐associated ketoacidosis have emerged as critical clinical entities. Although historically termed euglycemic diabetic ketoacidosis (EDKA), this term is often a misnomer since greater than half of the presentations can be associated with hyperglycemia, making SGLT2i‐associated ketoacidosis the preferred and more accurate terminology. Furthermore, this complication is not exclusive to diabetic populations; it has been reported in patients without diabetes receiving SGLT2i for heart failure or chronic kidney disease [2]. In T2D patients, the incidence of EDKA ranges from 0.16 to 0.76 events per 1000 patients per year [ 3]. It is estimated that SGLT2i increases the risk of DKA in T2D patients sevenfold [4]. The true incidence of SGLT2i‐associated ketoacidosis is frequently underestimated in the literature, with data indicating it can reach up to 4.9 events per 1000 patient‐years [5], underscoring its clinical significance. In critically ill patients, diagnosis can be complex due to the absence of marked hyperglycemia and other hemodynamic conditions that may mask the metabolic state; therefore, a high index of suspicion is required to prevent diagnostic delays.

## 2. Case Presentation

### 2.1. Case 1: Surgical Stress and Cardiac Surgery

A 65‐year‐old male with a recent diagnosis of T2D treated with empagliflozin/metformin (12.5 mg/850 mg BID) and dyslipidemia managed with atorvastatin (80 mg QD). He presented to the emergency department with typical anginal chest pain and worsening functional class over 3 weeks. Diagnostic workup included an electrocardiogram showing nonspecific repolarization changes and a transthoracic echocardiogram revealing segmental wall motion abnormalities; subsequent percutaneous coronary angiography confirmed triple‐vessel coronary artery disease. He was scheduled for coronary artery bypass graft (CABG) surgery 1 week later.

Complete revascularization was performed (LIMA to LAD, radial artery to OM, and two saphenous vein grafts) with a cardiopulmonary bypass time of 104 min and an aortic cross‐clamp time of 79 min. No intraoperative complications were reported. The patient was admitted to the intensive care and coronary care unit stable, without vasopressor requirements, and extubated.

Six hours after admission, he developed arterial hypotension, high anion gap metabolic acidosis with normal glucose levels (Table 1), and somnolence. Other causes of high anion gap metabolic acidosis were ruled out, including normal lactate levels; SGLT2i‐associated ketoacidosis was diagnosed based on the presence of urinary ketones and the use of empagliflozin until 24 h prior to surgery. Management was initiated with subcutaneous insulin and intravenous crystalloids, showing initial improvement and resolution criteria after 8 h. However, due to poor oral intake, ketoacidosis recurred 24 h after initial management. Treatment was escalated to intravenous rapid‐acting insulin infusion, crystalloids, and 10% dextrose solution, achieving definitive resolution at 48 h postdiagnosis. Clinical evolution was favorable thereafter, with no immediate surgical complications. He remained stable on the ward with capillary glucose levels between 110 and 180 mg/dL without insulin requirement. Discharge occurred 72 h postsurgery without SGLT2i, which was reinstated during follow‐up 3 weeks later.

**Table 1:** Laboratory results for Clinical Case No. 1.

Laboratory test	Initial (to the SGLT2i‐a KA diagnosis)	Follow‐up at 8 h	Follow‐up at 24 h	Follow‐up at 48 h (SGLT2i‐a KA resolution)
Blood glucose	133 mg/dL	147 mg/dL	171 mg/dL	167 mg/dL
Serum sodium	133.2 mmol/L	136.3 mmol/L	137.9 mmol/L	142.1 mmol/L
Serum potassium	3.4 mmol/L	4.0 mmol/L	4.9 mmol/L	3.4 mmol/L
Serum chloride	101 mmol/L	104 mmol/L	112 mmol/L	112 mmol/L
Urinary pH	5.0	5.0	5.0	6.5
Urinary glucose	2000 mg/dL	2000 mg/dL	2000 mg/dL	2000 mg/dL
Ketones in urine	80 mg/dL	80 mg/dL	80 mg/dL	80 mg/dL
Venous pH	7.29	7.38	7.15	7.46
Serum bicarbonate	12.2 mmol/L	17.7 mmol/L	5.9 mmol/L	22.8 mmol/L
Anion gap	21.0	18 mmol/L	22 mmol/L	8 mmol/L
Serum lactate	2.20 mmol/L	1.20 mmol/L	1.40 mmol/L	0.90 mmol/L

Abbreviation: SGLT2i‐a KA, Sodium‐glucose cotransporter 2 inhibitors‐associated ketoacidosis.

### 2.2. Case 2: Pharmacological Interaction (SGLT2i + GLP − 1)

A 45‐year‐old male with a 3‐year history of T2D, previously treated with insulin glargine (30 UI QD) and dapagliflozin/metformin (5 mg/1000 mg QD). During a follow‐up visit, treatment was adjusted to oral semaglutide (7 mg tablet) and empagliflozin (25 mg). Twenty‐four hours later, the patient developed nausea, hiccups, and oral intolerance, which worsened with vomiting. Upon admission, he exhibited mild agitation, Kussmaul breathing, tachycardia, a capillary refill time of 4 s, and clinical signs of severe dehydration.

Arterial blood gas revealed metabolic acidosis, a prolonged anion gap, normal glucose levels, and urinary ketones, confirming SGLT2i‐associated ketoacidosis and Stage 1 acute kidney injury (Table 2). SGLT2i was suspended, and management with crystalloids, rapid‐acting insulin infusion, and 10% dextrose solution was initiated. He was admitted to the intermediate care unit, where resolution was achieved within 24 h, and insulin glargine (15 UI QD) was started. The patient remained hemodynamically stable without vasopressors, with resolution of the kidney injury. He was discharged 48 h after admission, restarting dapagliflozin/metformin and insulin glargine.

**Table 2:** Laboratory findings for Clinical Case No. 2.

Laboratory test	Initial (to the SGLT2i‐a KA diagnosis)	Follow‐up at 8 h	Follow‐up at 24 h (SGLT2i‐a KA resolution)
Blood glucose	170 mg/dL	102 mg/dL	82 mg/dL
Serum sodium	139.2 mmol/L	139.4 mmol/L	140 mmol/L
Serum potassium	4.5 mmol/L	3.6 mmol/L	3.6 mmol/L
Serum chloride	102 mmol/L	109 mmol/L	108 mmol/L
Serum creatinine	1.52 mg/dL	—	0.89 mg/dL
BUN	23 mg/dL	—	13 mg/dL
Urinary pH	5.0	5.0	5.0
Urinary glucose	2000 mg/dL	2000 mg/dL	2000 mg/dL
Ketones in urine	80 mg/dL	80 mg/dL	80 mg/dL
Venous pH	7.27	7.39	7.40
Serum bicarbonate	13.30 mmol/L	15.70 mmol/L	19.80 mmol/L
Anion gap	26.0	17 mmol/L	17 mmol/L
Serum lactate	3.00 mmol/L	0.80 mmol/L	0.90 mmol/L

Abbreviations: BUN, Blood Urea Nitrogen; SGLT2i‐a KA, Sodium‐glucose cotransporter 2 inhibitors‐associated ketoacidosis.

## 3. Discussion

Predisposing factors for DKA, beyond SGLT2i use, include acute stress (pulmonary embolism, acute inflammatory diseases, myocardial infarction) and specific metabolic states such as hypertriglyceridemia, abrupt dietary changes, fasting, carbohydrate‐restricted diets, and hypermetabolic states like pregnancy, malignancy, or severe infections [6, 7]. A multicenter cohort study by Ata et al. reported an EDKA prevalence of 0.25% associated with SGLT2i, with infection being the most common trigger (16%) [8]. Although some studies cite dapagliflozin as the most frequent associate, others report a higher association with canagliflozin [9].

In Case 1, acute stress from CABG surgery served as the precipitant. Major surgical procedures, especially with prolonged preoperative fasting, trigger an increase in counter‐regulatory hormones (glucagon, catecholamines, and cortisol), enhancing lipolysis and ketogenesis, which is exacerbated by SGLT2i‐induced glucosuria. The incidence of EDKA in cardiac surgery varies from 1%–15%, with insufficient drug suspension time identified as a key risk factor [10]. McMann et al. reported that SGLT2i‐associated ketoacidosis can occur up to 15% specifically in CABG [11]. Consequently, it is recommended to suspend SGLT2i at least 3–5 days before surgery [9]. A key management pitfall highlighted in this case is the initial reliance on subcutaneous insulin protocols. Although subcutaneous insulin may be appropriate for mild, stable cases of classic DKA [12], SGLT2i‐associated ketoacidosis in the postsurgical setting presents a distinct physiological challenge. As previously described in characterizations of treatment responses for SGLT2i‐associated ketoacidosis [13], the persistent circulating drug levels continue to drive glucosuria and promote lipolysis over an extended period. This prolonged pathophysiological drive explains the rebound ketosis observed in our patient at 24 h when subcutaneous insulin levels waned. The increased counter‐regulatory hormones and persistent glucosuria require a more aggressive and titratable approach. Our patient′s early recurrence demonstrates that subcutaneous delivery may fail to provide the consistent serum levels needed to shut down hepatic ketogenesis in a hypermetabolic state.

Case 2 illustrates a different trigger: therapeutic modification. Although semaglutide (a GLP‐1 receptor agonist) is generally considered safe regarding ketoacidosis risk, the underlying mechanism mimics the state seen during restrictive bowel preparations or colonoscopies where SGLT2i‐associated ketoacidosis was initially recognized [14]. The marked reduction in caloric and carbohydrate intake induced by semaglutide reduces glucose‐stimulated insulin secretion. This drastically lowers the insulin‐to‐glucagon ratio, unleashing uninhibited fatty acid metabolism and accelerating hepatic ketone production. Therefore, a stepped management approach for T2D is crucial, avoiding excessive carbohydrate restriction in patients starting GLP‐1 agonists with concomitant SGLT2i use [15]. Finally, regarding the clinical decision to restart SGLT2i in Case 1, it must be explicitly emphasized that the definitive safety of reintroduction following an episode of ketoacidosis has not been demonstrated in clinical trials. Although it is routinely performed on a case‐by‐case basis in clinical practice due to metabolic or cardiovascular needs, it requires absolute caution and close patient education.

A limitation in these cases was the reliance on urinary ketones for diagnosis. Urinalysis measures acetoacetate rather than beta‐hydroxybutyrate, the predominant ketone body in ketoacidosis. This introduces several pitfalls: first, there is an inherent time delay in urinary ketone elevation during onset; second, acetoacetate remains persistently elevated in urine long after the metabolic crisis has resolved, giving a misleading impression of worsening ketosis; third, SGLT2i enhance the renal reabsorption of ketone bodies, which may paradoxically lower urinary measurements and result in a missed diagnosis. In line with the American Diabetes Association consensus statement on hyperglycemic crises, the use of finger‐prick point‐of‐care capillary ketone assessment must be prioritized in the ICU to avoid these diagnostic errors [12].

Furthermore, a critical lesson for the ICU team is the early implementation of 10% dextrose solution. The goal of therapy in SGLT2i‐associated ketoacidosis is not primarily glucose lowering, but the suppression of ketone production. Providing prolonged 10% dextrose infusions allows the administration of sufficient IV insulin doses to improve the metabolic abnormalities without risking severe hypoglycemia. Early recognition is paramount for timely diagnosis and appropriate management.

## 4. Conclusion

SGLT2i‐associated ketoacidosis remains a diagnostic challenge in critical care due to its normoglycemic presentation. A high index of suspicion is required in postoperative patients or those with recent changes in combination therapies (SGLT2i + GLP − 1). Implementing 72‐h preoperative suspension guidelines and early initiation of intravenous insulin protocols with glucose supplementation are essential to improving outcomes.

## Funding

No funding was received for this manuscript.

## Disclosure

The authors have disclosed no financial relationships relevant to this article. This commentary does not contain a discussion of an unapproved/investigative use of a commercial product/device.

## Conflicts of Interest

The authors declare no conflicts of interest.

## Supporting information


**Supporting Information** Additional supporting information can be found online in the Supporting Information section.

## Data Availability

The data that support the findings of this study are available on request from the corresponding author. The data are not publicly available due to privacy or ethical restrictions.
